# Shedding Light on the Formation and Structure of Kombucha Biofilm Using Two-Photon Fluorescence Microscopy

**DOI:** 10.3389/fmicb.2021.725379

**Published:** 2021-08-04

**Authors:** Thierry Tran, Cosette Grandvalet, Pascale Winckler, François Verdier, Antoine Martin, Hervé Alexandre, Raphaëlle Tourdot-Maréchal

**Affiliations:** ^1^UMR Procédés Alimentaires et Microbiologiques, AgroSup Dijon, Université de Bourgogne Franche-Comté, Dijon, France; ^2^INRA, INSERM, Dimacell Imaging Facility, AgroSup Dijon, Université de Bourgogne Franche-Comté, Dijon, France; ^3^Biomère, Paris, France

**Keywords:** kombucha, biofilm, cellulose, interaction, two-photon fluorescence microscopy

## Abstract

Kombucha pellicles are often used as inoculum to produce this beverage and have become a signature feature. This cellulosic biofilm produced by acetic acid bacteria (AAB) involves yeasts, which are also part of the kombucha consortia. The role of microbial interactions in the *de novo* formation and structure of kombucha pellicles was investigated during the 3 days following inoculation, using two-photon microscopy coupled with fluorescent staining. Aggregated yeast cells appear to serve as scaffolding to which bacterial cellulose accumulates. This initial foundation leads to a layered structure characterized by a top cellulose-rich layer and a biomass-rich sublayer. This sublayer is expected to be the microbiologically active site for cellulose production and spatial optimization of yeast–AAB metabolic interactions. The pellicles then grow in thickness while expanding their layered organization. A comparison with pellicles grown from pure AAB cultures shows differences in consistency and structure that highlight the impact of yeasts on the structure and properties of kombucha pellicles.

## Introduction

Starting as a traditional fermented beverage, kombucha is nowadays getting increasing popularity as a bottled beverage produced at an industrial scale (Kim and Adhikari, 2020). Nevertheless, the image that one generally has from kombucha is rather those of a kitchen jar containing the fermented sugared tea in which floats a macroscopic cellulosic mat that bears different names such as “pellicle,” “mother,” “tea fungus,” or more recently “SCOBY” (symbiotic culture of bacteria and yeasts) (Greenwalt et al., 2000; Jayabalan et al., 2014; Villarreal-Soto et al., 2018). Indeed kombucha is a tea-based beverage engendered by a microbial consortium including yeasts, acetic acid bacteria (AAB), and, in some cases, lactic acid bacteria (Dufresne and Farnworth, 2000; Jayabalan et al., 2014; Villarreal-Soto et al., 2018). The metabolic interactions between yeasts and AAB allow the sugared tea infusion to be transformed into a sparkling sour beverage, similar to a soda with often lower sugar content (Blanc, 1996; Chen and Liu, 2000; Jayabalan et al., 2010; Tran et al., 2020a). Kombucha is produced by inoculating a sugared tea infusion with a kombucha culture using its broth, the pellicle, or both. The acidification of the broth occurs in an open vessel to ensure access to oxygen by microorganisms, acetic acid bacteria in particular (Tran et al., 2020b), while a new pellicle forms at the surface. Sucrose is converted by yeasts into monosaccharides (glucose and fructose) and ethanol through invertase activity and alcoholic fermentation, respectively. These compounds serve as substrates for AAB, with the production of gluconic and acetic acids through oxidative metabolism (Liu et al., 1996; Jayabalan et al., 2014; Villarreal-Soto et al., 2018; Lynch et al., 2019; Tran et al., 2020a). Natural carbonation can then be performed by bottling after discarding the pellicle (Tran et al., 2020a,b). As previously stated, kombucha pellicle is used as a solid inoculum to be added to a fresh batch of sugared tea that could be shared and spread, hence the large diffusion of homemade kombucha across the world (Zagrabinski, 2020a,b). The cellulosic structure is formed by AAB and can also be grown in pure AAB cultures (Iguchi et al., 2000; Gullo et al., 2018).

*Komagataeibacter xylinus* is the model bacteria used to elucidate the formation mechanism of cellulose by AAB. *K. xylinus* can use a range of compounds to produce cellulose, including hexoses, glycerol, pyruvate, and dicarboxylic acids. These compounds are involved in gluconeogenesis, leading indirectly to the cellulose precursor: uridine diphosphate glucose. The polymerization of glucose units bound by β1→4 linking using this precursor is catalyzed by cellulose synthase (Ross et al., 1991). This transmembrane enzyme of 400–500 kDa can be seen as 50–80 pores organized spatially in row along the cell for the extrusion of cellulose. Cellulose synthase takes uridine diphosphate from the cytosol and extrudes 2–4 nm protofibrils into the extracellular medium (Iguchi et al., 2000; Chawla et al., 2009). Protofibrils associate into ribbon-shaped microfibril of 80 × 4 nm (Chawla et al., 2009). Under static conditions, as it is the case for kombucha, the result is a cellulosic mat floating at the air/liquid medium interface (Iguchi et al., 2000; Czaja et al., 2004). Intensive research was carried out because of the remarkable rheological properties of bacterial cellulose, which is purer than plant cellulose and can potentially be used as a biocompatible material for health applications (Iguchi et al., 2000; Esa et al., 2014; Zhu et al., 2014).

Cellulosic pellicles hosting microorganisms from kombucha and vinegar can be considered as biofilm despite their macroscopic scale. Biofilms are aggregates of microorganisms embedded in a self-produced matrix of extracellular polymeric substances (Vert et al., 2012), and several research works include sessile microbial structures without a solid surface in the definition of a biofilm (Zara et al., 2005; Alhede et al., 2011; David-Vaizant and Alexandre, 2018). These resistance forms limit the diffusion of toxins, the impact of brutal changes and extreme conditions in environmental parameters such as pH, temperature, or hygrometry, and facilitates the access to nutrients and microbial communication *via* quorum-sensing signalization in some bacterial species (Davies, 1998; Carlier et al., 2015; Flemming et al., 2016). It has been hypothesized that AAB formed floating cellulose mats to facilitate their access to oxygen as obligate aerobes and that the extracellular matrix could protect them against ultraviolet rays, other microorganisms, and heavy metals (Schramm and Hestrin, 1954; Iguchi et al., 2000). It has also been suggested that AAB cellulosic biofilm was the matrix supporting cellular communication, as it has been observed with model bacteria (Cooper et al., 2014; Gullo et al., 2018). Several of those putative functions of cellulosic biofilms are suspected to be applicable to kombucha pellicle and to play an important role in microbial interaction (May et al., 2019). Very little investigation has been carried out about the kombucha pellicle, especially through the lens of spatial organization and microbial interactions (Mayser et al., 1995; Goh et al., 2012; Zhu et al., 2014; Coton et al., 2017; Sharma and Bhardwaj, 2019; Betlej et al., 2020). The study of Coton et al. (2017) reported differences in microbial composition in the proportion of genera between the liquid phase and the biofilms for yeasts and bacteria using a non-culture dependent method, thus highlighting the impact of two different environments in terms of planktonic and sessile state or oxygen and nutrient access on microbial dynamics (Coton et al., 2017). A recent study showed that the synthetical consortia made of one yeast and one AAB species isolated from the same kombucha culture were able to perform the elaboration of kombucha-like beverages without the presence of a consistent biofilm, especially during the acidification phase (Tran et al., 2020a).

This pioneer study aims to investigate the relationship binding the structure of kombucha biofilm with microbial interactions and related functions, namely, at the early-stage biofilm formation. Besides the macroscopic observations and chemical and microbiological analyses, this study was based on two-photon microscopy. This technique is non-invasive and allows better penetration in thick samples compared to confocal fluorescence microscopy and is therefore adapted to the study of kombucha cellulosic pellicles (Helmchen and Denk, 2005). Two-photon microscopy can provide not only visual information on the structure of an object but also about its composition when coupled with fluorescence staining. The staining of polysaccharides and nucleic acids was used to visualize the cellulose structure and cells according to their physiological state.

## Materials and Methods

### Generation of Kombucha Pellicle Samples

The production of sugared black tea and traditional kombucha was carried out according to a previous work (Tran et al., 2020a). Briefly, after steeping 1% (m/v) of black tea, 60 g L^–1^ of sucrose was dissolved and left to cool before inoculation with 12% (v/v) of 14-day-old black tea kombucha broth only; no pellicle was added so that the *de novo* formation of a pellicle could be investigated. To ensure a physiological state of microorganisms comparable to regular industrial-scale production, a primary inoculum was produced using the same procedure, except that it was inoculated using a mother culture. The mother culture is a kombucha culture provided by Biomère (Paris, France) that was refreshed monthly with sugared black tea. All cultures were carried out in triplicate in 123 ml Boston flasks with a specific interfacial surface of 0.01 cm^–1^. Bottlenecks were loosely covered with tin fold to allow gas exchanges. Incubation time was 3 days at 26°C in static conditions. The observation of the very first 3 days of biofilm formation was chosen to ensure low biofilm thickness and focus on the early formation steps.

### Generation of Pellicle Samples From Pure Acetic Acid Bacteria Culture

To investigate the role of yeasts in the formation and structure of pellicles, kombucha biofilms were compared with pure AAB biofilm in sugared black tea. Modified sugared black tea was inoculated with two strains of AAB isolated from black tea kombucha, with sucrose being substituted with 1:1 amount of glucose and fructose. *Acetobacter indonesiensis* and *Komagataeibacter saccharivorans* isolated from kombucha (Tran et al., 2020a) were inoculated at a rate of 1 × 10^5^ CFU ml^–1^ from a 3-day preculture in liquid De Man, Rogosa and Sharpe (MRS) medium at 28°C using the same procedure as in a previous work (Tran et al., 2020a). Cultivation then occurred in the same conditions as the kombucha samples, but with an extended incubation duration of 14 days—the time required under these conditions for pellicle production. Consequently, the comparison will focus on the structure of the biofilm at endpoint and purposely avoid any dependence of AAB regarding yeast invertase activity by having monosaccharides available in the medium.

### Macroscopic Observation and Chemical Analyses

A macroscopic observation of pellicles was made from the time of inoculation (D0), during the next 3 days of production (D1, D2, and D3) for kombucha, and after 14 days for pure AAB cultures.

Variations of fresh pellicle weight were determined with biological triplicates that were sacrificed for each measurement. The determination of dry weight content was also made for each measurement point in triplicates using gravimetric methods after treating the samples at 102°C in an oven for 24 h.

The samples were kept frozen at −20°C and were centrifuged prior to chemical analyses (3,000 × *g*, 15 min, 10°C). After thawing, the pH was determined using a Mettler Toledo Five Easy pH meter coupled with a LE498 probe. Total acidity was determined by titration with 1 N NaOH and 0.2% phenolphthalein as the color indicator (OIV, 2009).

### Microbiological Analyses of Kombucha

A microbial composition analysis of the kombucha culture was performed by isolating 12 yeast colonies per morphotype, and eight bacteria colonies were isolated on Wallerstein Lab (WL) agar medium (Green and Gray, 1951) and MRS agar medium (pH 6.2), respectively. WL agar allows the discrimination of yeasts according to the shape and color of the colonies (Pallmann et al., 2001). The isolates were re-streaked on appropriate agar and incubated at 28°C for 5 days for fluorescence microscopic observation and DNA extraction. DNA extraction, amplification, and sequencing were performed according to a previous work (Tran et al., 2020a). The 26S and 16S PCR were used to amplify DNA extracts from yeasts and bacteria, respectively (Maoura et al., 2005; Wang and Qian, 2009). For yeasts, the 26S rDNA region ribosomal non-transcribed spacer 2 (NTS 2) was amplified using the following primers: NL1 (50-GCATATCAATAAGCGGAGGAAAAG-30) and NL4 (50-GTCCGTGTTTCAAGACGG-30) (Maoura et al., 2005). For bacteria, the 16S ribosomal DNA was amplified using the following primers: E517F (50-GCCAGCAGCCGCGGTAA-30) and E106R (50-CTCACGRCACGAGCTGACG-30) (Wang and Qian, 2009). Sanger sequencing of both strands was performed on amplified DNA by Genewiz^®^ (Leipzig, Germany) using corresponding primers for yeasts and bacteria. The sequences were analyzed using Geneious R7 (version 7.1.5) and Basic Local Alignment Search Tool of NCBI^1^, thus returning genus and species names. The identities were determined based on the lowest *E*-value.

### Fluorescence Imaging Microscopy

Two-photon imaging microscopy was performed to achieve 2D and 3D (z-stacks) representations of the kombucha pellicle. The images were collected on a Nikon A1-MP scanning microscope equipped with a Plan APO IR × 60 objective (NA, 1.27; Water Immersion, Nikon) at a scanning speed of one frame per second. An IR laser (Chameleon, Coherent) was used to provide excitation at 800 nm. Fluorescence emission was collected on four detection channels: FF01-492/SP (400–492 nm), FF03-525/50 (500–550 nm), FF01-575/25 (563–588 nm), and FF01-629/56 (601–657 nm) (Semrock). The images provided in the manuscript are obtained by merging these four detection channels without any other spectral selection. All images were processed in the same way to optimize contrast between channels.

The pellicles were simply withdrawn from the liquid surface using pliers and placed between a glass slide and a coverslip after labeling. The pellicle fragments at D1 were withdrawn at the surface of the liquid using an enlarged pipet tip. A drop of 1 μl of each fluorescent label was deposed directly on the pellicle. Cellulose was labeled using 1 mM calcofluor from Merck (Darmstadt, Germany) (Haigler et al., 1980). Yeasts and bacteria were labeled using propidium iodide (PI) and SYTO9 (green fluorescent nucleic acid stain) simultaneously, a widely used mix for cell viability assay (Stiefel et al., 2015; Kirchhoff and Cypionka, 2017). The nucleic acids of viable cells were stained using 5 mM SYTO9 from Thermo Fisher Scientific (Carlsbad, United States), which possesses a green fluorescence (emission maximum at 503 nm). Nucleic acids in dead or damaged cells and in extracellular medium were stained using 0.2 μM propidium iodide in distilled water from Merck (Darmstadt, Germany). Propidium iodide possesses a stronger affinity to nucleic acids than SYTO9 and possesses a red fluorescence (emission maximum at 636 nm). Unspecific fluorescent labeling was made using fluorescein from Merck (Darmstadt, Germany) that is used at D1 to strengthen the visualization of cells at a concentration of 1 nM and possesses a green fluorescence; therefore, it was not used in combination with SYTO9. Different control tests have been carried out. No autofluorescence appeared on the images made with the microscope settings detailed above. The individual labeling of each fluorochrome, as well as every combination of simultaneous labeling, has been tested. The presence of SYTO9 aggregates has been observed and could be due to the low pH value (below 4) of the liquid phase in which the pellicle forms. It has also been observed that the addition of propidium iodide alone caused a slight outline of yeasts, and this signal got stronger with increasing concentration.

## Results

### Visual and Physical Chemical Parameters of Pellicle Formation During Kombucha Elaboration

**FIGURE 1:**
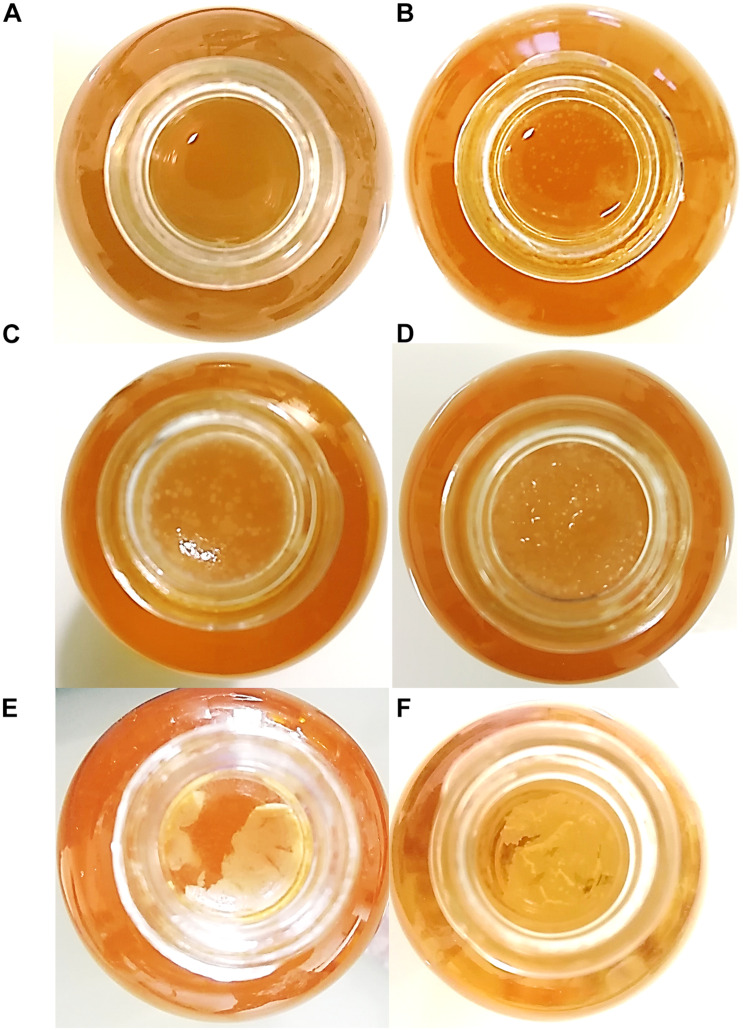
Macroscopic aspect of pellicles from top view in a Boston bottle. Kombucha-grown pellicles in sugared tea at D0 **(A)**, D1 **(B)**, D2 **(C)**, and D3 **(D)**. Pellicles grown by pure acetic acid bacteria culture in modified sugared tea at D14 **(E)** by *Acetobacter indonesiensis* and **(F)** by *Komagataeibacter saccharivorans*. The diameter of the bottlenecks and pellicles is 10 mm.

The macroscopic aspect of kombucha pellicles was followed at its early stages of formation after inoculation with broth only and no pellicle (Figure 1). Starting from a plain liquid suspension in sugared black tea at D0 (Figure 1A), biofilm fragments could be observed floating at D1 (Figure 1B). A consistent pellicle was formed at D2 and was very smooth and flexible yet consistent and could be easily manipulated using pliers (Figure 1C). On the next and final day of the experiment, the pellicle got more rigid and looked rougher on the upper surface (Figure 1D). Pellicles grown from pure AAB cultures in modified sugared tea were produced. Firstly, their growth rate was much longer since no floating particle could be seen before 7 days of incubation, thus preventing any comparison at D3. The present photographs were taken after 14 days of incubation (Figures 1E,F). The aspect of these pellicles was very different from the ones produced by the kombucha culture with a smooth aspect, low thickness, and poor consistency, preventing any manipulation by breaking into smaller fragments. This could result from the difference in the inoculation method, with only addition of cells in the case of pure cultures as opposed to the addition of 12% (v/v) of kombucha from a previous batch in the other case. The resources of nitrogen, quantitatively and qualitatively, were probably different, and the absence of ethanol could play a role in the rate of cellulose production (Nguyen et al., 2008; Mamlouk and Gullo, 2013; Betlej et al., 2020). Another hypothesis would lie in the presence of microbial interactions that come with the use of a consortium.

During kombucha elaboration, the pH value of sugared tea dropped from 6.66 to 4.27 following the addition of the inoculum (previous batch), with a total acidity of 86 ± 4.3 meq L^–1^ in the elaborate product (Table 1). The pH value decreased between D0 and D3 until 3.67 ± 0.02, and the total acidity increased from 7.7 ± 1.0 to 19.7 ± 1.3 meq L^–1^, proof that organic acid production, the central process of kombucha elaboration, has occurred. The fresh and dry weights increased significantly between D2 and D3 by around threefold, while the dry weight content did not change significantly (10% average). This value was reported for pellicles grown by AAB, and it is supposed that the dry weight rate measured is greatly influenced by microbial biomass rather than the cellulose itself (Schramm and Hestrin, 1954).

**TABLE 1:** Physical and chemical parameters of liquid phase and pellicle during kombucha elaboration (*n* = 3, average ± confidence interval with α = 0.05).

Sample	pH_liquid_	Total acidity_liquid_ (meq L^–1^)	Fresh weight_pellicle_ per tea volume (mg L^–1^)	Dry weight_pellicle_ per tea volume (mg L^–1^)	Dry weight content_pellicle_ (%)
Sugared black tea	6.66 ± 0	<1	Not applicable		
Inoculum	2.75 ± 0	86 ± 4.3			
D0 (after inoculation)	4.27 ± 0	7.7 ± 1.0			
D1	3.92 ± 0.07	14.7 ± 2.8			
D2 (samples 1, 2, and 3)	3.84 ± 0.06	17.7 ± 0.5	178.7 ± 115.3	16.5 ± 2.8	9.3 ± 1.6
D3 (samples 4, 5, and 6)	3.67 ± 0.02	19.7 ± 1.3	462.9 ± 446.3	51.7 ± 6.4	11.2 ± 0.4

### Morphology and Identification of Yeasts and Bacteria From Kombucha Consortium

Cultures of the liquid-phase samples at D0 on differential and selective agar media allowed the quantification of yeast and bacteria populations. The species identities obtained by biomolecular method were characterized according to colony morphotype and microscopic aspect (Table 2).

**TABLE 2:** Identification and description of yeast and bacteria in the liquid phase at D0.

Microorganism	PCR type	*E*-value	Colony morphotype
**Yeasts**		0	
*Brettanomyces bruxellensis*	26S	0	Small white colonies appearing after the other yeast colonies (3 days after)
*Hanseniaspora valbyensis*	26S	0	Green colonies
*Saccharomyces cerevisiae*	26S	0	Large white colonies
**Bacteria**			
*Acetobacter papayae*	16S	0	Translucid colonies
*Gluconacetobacter takamatsuzukensis*	16S	<0.001	Iridescent aspect when directly exposed to light

As reported previously (Tran et al., 2020a), the identity of yeast colonies grown on WL agar medium was easy to determine based on their aspect and the time of apparition. This allowed the quantification of yeast subpopulations according to the species. As an example, *Brettanomyces bruxellensis* colonies appeared after several days and are small and white, whereas *Hanseniaspora valbyensis* colonies appeared quicker and are green. This approach could not be applied to acetic acid bacteria, whose colonies had very similar aspects. The iridescence observed when light is directly projected on these colonies might be due to a diffraction phenomenon caused by the crystallinity of bacterial cellulose (Goh et al., 2012). At D0, the total yeast population was larger than the total bacteria population with 4.6 × 10^5^ and 1.6 × 10^5^ CFU ml^–1^, respectively. The yeast population was dominated by the species *H. valbyensis* with 4.1 × 10^5^ CFU ml^–1^. The second largest yeast population was *B. bruxellensis* with 5 × 10^4^ CFU ml^–1^, and finally the lowest population detected was *Saccharomyces cerevisiae* with 2.7 × 10^3^ CFU ml^–1^. Since the pellicle formed from the inoculum sample was not added to the fresh sugared tea, those three yeast species were expected to be part of the newly formed biofilm together with the two species of acetic acid bacteria identified: *Acetobacter papayae* and *Gluconacetobacter takamatsuzukensis*. Focus was put on acetic acid bacteria, and therefore no anaerobic culture was carried out. Consequently, the isolation of aero anaerobic lactic acid bacteria might have been overlooked.

**FIGURE 2:**
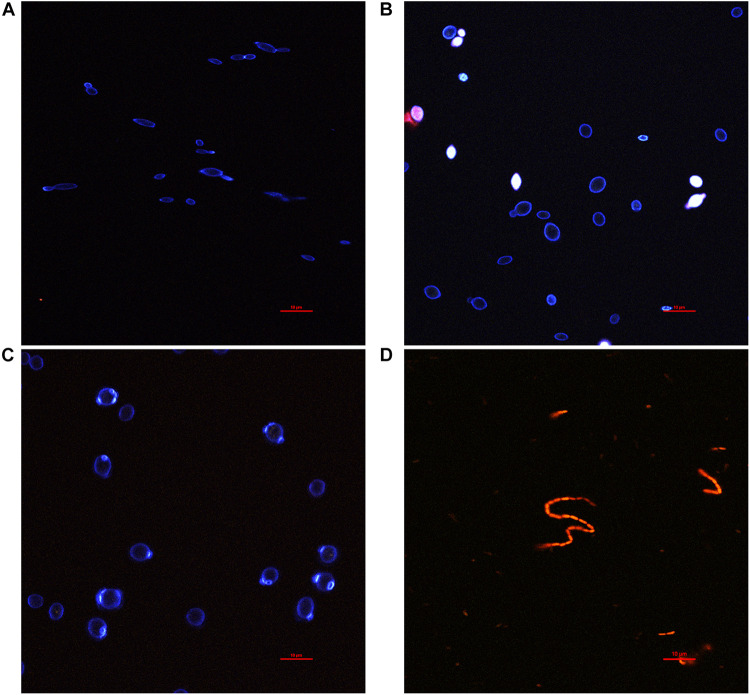
Fluorescence microscopy observation of yeasts and bacteria isolates from agar plate cultures stained with calcofluor (blue), propidium iodide (red), and fluorescein (green) channels are mixed. **(A)** *Brettanomyces bruxellensis*, **(B)** *Hanseniaspora valbyensis*, **(C)** *Saccharomyces cerevisiae*, and **(D)** *Acetobacter* sp.

At a microscopic scale, distinct cell morphologies could be characterized from colonies grown on agar plates using two-photon microscopy and fluorescent labeling (Figure 2). Calcofluor labels polysaccharides, including cellulose or cell wall compounds, and PI stains nucleic acids when not blocked by non-permeable functional membranes.

Calcofluor staining (blue) highlighted the yeast cell walls, whereas no staining could be observed for AAB (Figure 2). The bacteria were instead fully stained with PI, which is a sign of a permeable cell membrane. It should be noted that staining inconsistencies have been reported regarding the PI staining of bacteria, leading to false-positive staining regarding the viability of cells (Kirchhoff and Cypionka, 2017). On the contrary, PI staining seemed to discriminate better permeable and non-permeable yeast cells with complete staining of the cytosol of permeable cells or even leaking intracellular material into the outer medium. The yeast cell morphologies were very different across species, whereas it was very consistent between AAB species. Images of the latter AAB species were not displayed as they were redundant with the aspect observed for *Acetobacter* sp. *B. bruxellensis* was characterized by high polymorphism, with cell size ranging between 3 and 10 μm and diverse shapes ranging from a “rice grain”-like shape to elongated or round (Figure 2A). Bud scars were also very visible. *H. valbyensis* cells were more homogenous, with an average cell size between 5 and 10 μm and characteristic polar budding leading to round or “lemon”-like shapes without visible bud scars (Figure 2B). The *S. cerevisiae* cells were about 7–10 μm and recognizable by their regular round shape with visible circular bud scars (Figure 2C). Finally, the AAB cells were about 1 μm long and were mostly organized by pairs, although chains of more than 30 cells could be observed (Figure 2D).

### Observation of Kombucha Pellicle Formation by Fluorescence Microscopy

**FIGURE 3:**
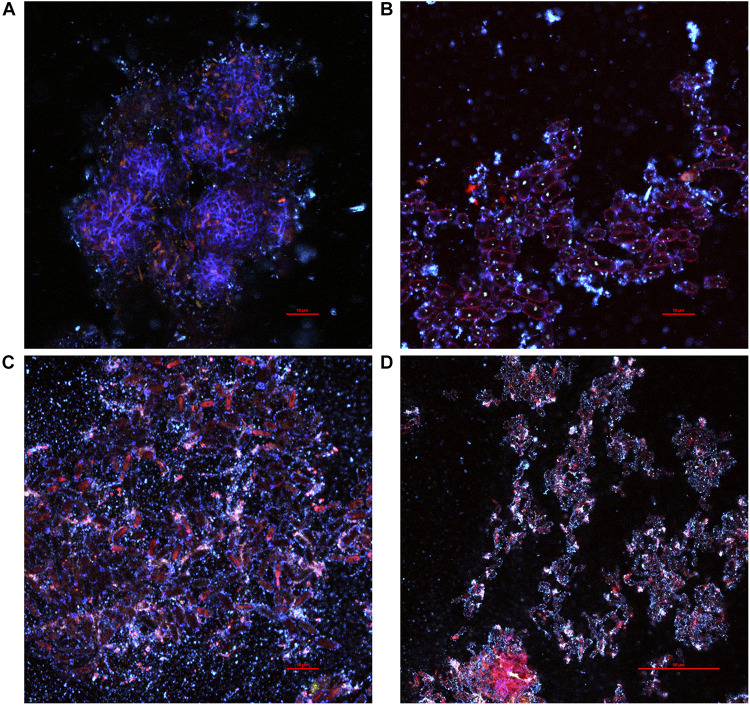
Various pellicle fragments observed at D1 during kombucha elaboration sampled from the surface of the liquid phase using two-photon microscopy, featuring mainly **(A)** bacteria, **(B,C)** yeasts, or both **(D)**. Fluorescence labeling of cellulose with calcofluor (blue) and of nucleic acids outside or inside of damaged cells with propidium iodide (red) and unspecific labeling with fluorescein (green).

Due to low cell density at D0, no meaningful image could be acquired by two-photon microscopy. The floating biofilm fragments observed at D1 (Figure 1B) could be withdrawn and observed (Figure 3). Bacteria stained by PI (red) were spotted to be aggregated in globular filamentous structures of cellulose stained by calcofluor (blue) (Figures 3A,D; at the bottom of the image). Other structures involved what appeared to be yeasts, based on their size (Figures 3B–D). Cells with their cytosol stained by PI were supposedly non-viable, while cells that were simply outlined by PI were supposedly viable. Yeast cells appeared aggregated, but it is unclear whether yeast cells were simply aggregated or if they were in pseudo-mycelium form. All around those aggregates floated smaller globular cellulose particles that appeared to accumulate at the surface of yeast aggregates, thus increasing their cohesion. The diversity in size and shape of the observed entrapped yeast cells could point at *B. bruxellensis* or the association of different species. It can thus be hypothesized that the further aggregation process, facilitated by the entrapping action of accumulating cellulose produced by AAB, should lead to the formation of a consistent pellicle as observed at D2 (Figure 1C).

**FIGURE 4:**
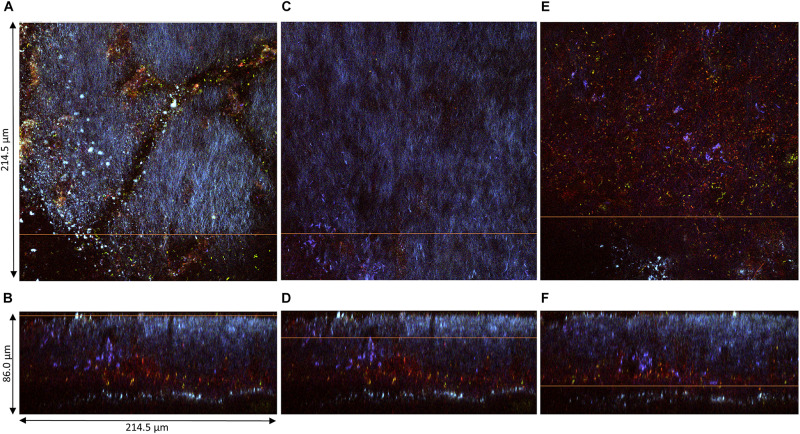
Pellicle (sample 1) observed at D2 during kombucha elaboration using two-photon microscopy. Cross-section of the **(A)** top region, **(C)** middle region, and **(E)** bottom region, with the orange line defining the position of the longitudinal section (dimensions: 214.5 × 214.5 μm). Longitudinal sections **(B,D,F)**, with the orange line defining the position of the corresponding cross-section (214.5 × 86.0 μm). Fluorescence labeling of cellulose with calcofluor (blue), of permeable cells and external nucleic acids with propidium iodide (red), and of viable cells using SYTO9 (green).

**FIGURE 5:**
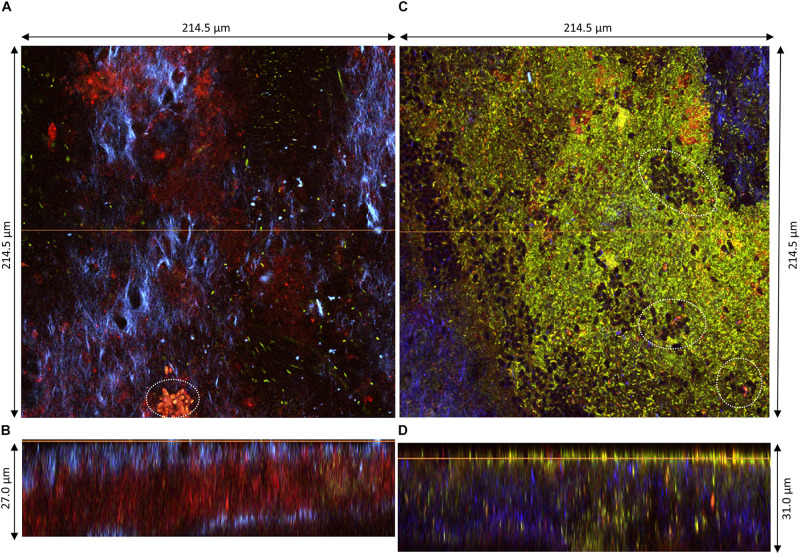
Pellicles observed at D2 during kombucha elaboration using two-photon microscopy. Longitudinal sections of **(A)** sample 2 (214.5 × 27.0 μm) and **(C)** sample 3 (214.5 × 31.0 μm), with the orange line defining the position of the corresponding cross-section **(B,D)**, respectively. Cross-sections of the top region of **(B)** sample 2 and **(D)** sample 3, with the orange line defining the position of the longitudinal section (dimensions: 214.5 × 214.5 μm). Fluorescence labeling of cellulose with calcofluor (blue), of permeable cells and external nucleic acids with propidium iodide (red), and of viable cells using SYTO9 (green). White dotted circles highlight examples of yeast microcolonies.

The pellicles produced at D2 (samples 1, 2, and 3) could be observed along their *z*-axis (thickness). Based on acquired z-stacks (Figures 4, 5), the thickness of pellicles was superior to 25 μm. The penetration depth of two-photon microscopy is dependent on the material analyzed for a given laser power. The maximal penetration was obtained for sample 1 (Figure 4). Fluorescent staining highlighted the presence of different layers: a thick top layer and a thin bottom layer with a predominance of cellulose and a middle layer heavily stained by PI (Figures 4B,D,F). The surface of the top layer consisted in filamentous cellulose in which bacteria stained by SYTO9 (green) dwell (Figure 4A). Under the immediate surface, the upper area mainly consists in cellulose organized in fibrils of different sizes (Figure 4C). The middle layer stained by PI hosted viable bacterial cells arranged individually or in chains surrounded by biomass stained by PI responsible for the overall red fluorescence of the layer (Figure 4E). Because of the quality of the staining and the laser power attenuation caused by the depth, it is difficult to precisely determine the nature of this biomass. However, it can be expected to be cell debris, potentially remaining of yeast cell walls.

The structures of samples 2 and 3 were also organized in layers but showed dissimilarities (Figure 5). The middle PI-stained layer was thicker in sample 2 (Figure 5B) and, on the contrary, was not present in sample 3 (Figure 5D). The surface of the top layer hosted in all cases viable and non-viable cells dwelling in cellulose fibrils (Figures 5A,C). Yeast microcolonies could be seen at the bottom of Figures 5A,C (white dotted circles). Non-viable yeast cells were stained with PI, with the whole cytosol stained, and viable cells show a slight nuclear stain with SYTO9. On the same plane as the yeast microcolony on Figure 5A, a PI-stained biomass belonging to the middle layer appeared, which points rather at remaining yeast cell walls rather than whole cells. The top layer surface of sample 3 exhibited numerous viable and fewer non-viable bacteria cells and unstained yeast cells (black holes) (Figure 5C). Issues with fluorescence labeling of non-model bacteria using a SYTO9/PI combination were reported, and they may also be applicable to non-model yeasts (Stiefel et al., 2015). Another limitation dwells in the difficulty to control the stain concentration at each location of the solid biofilm. Overall, the structure of pellicles at D2 seemed very variable.

**FIGURE 6:**
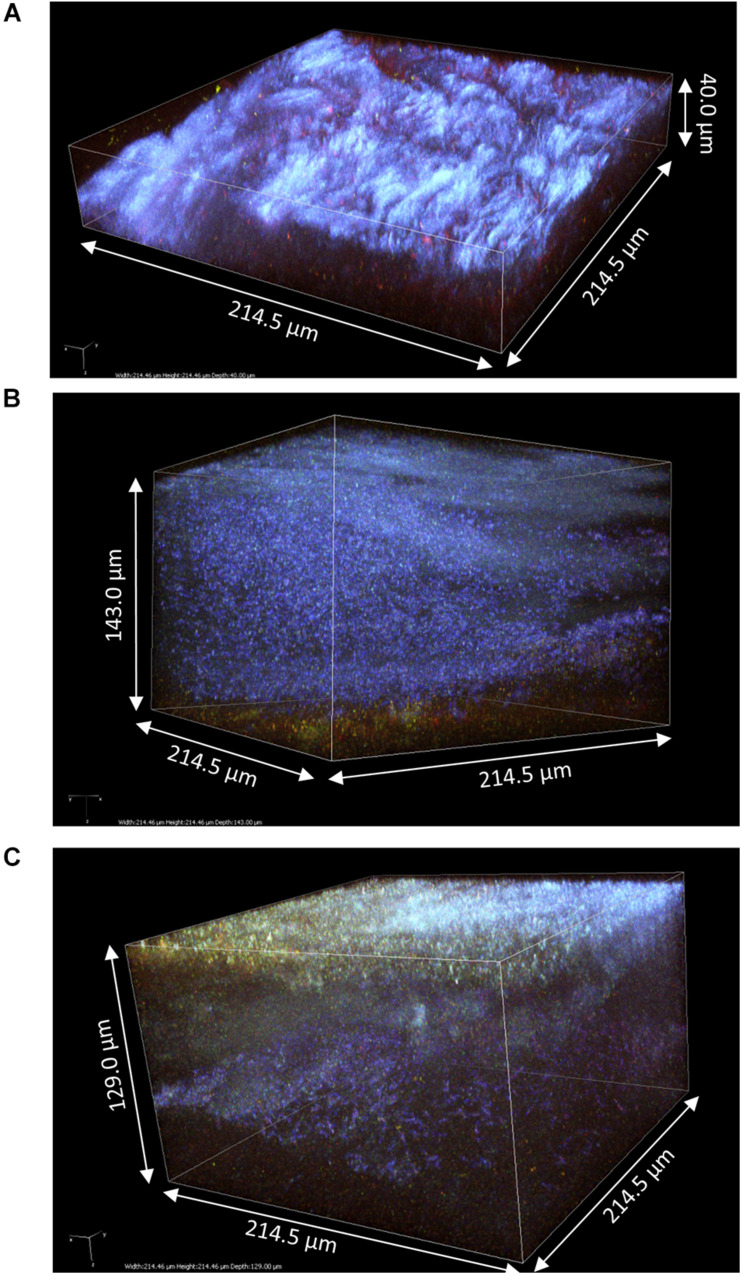
3D modeling of z-stack of the upper region of pellicles observed at D3 during kombucha elaboration using two-photon microscopy: **(A)** sample 4 (214.5 × 214.5 × 40.0 μm), **(B)** sample 5 (214.5 × 214.5 × 143.0 μm), and **(C)** sample 6 (214.5 × 214.5 × 129.0 μm). Fluorescence labeling of cellulose with calcofluor (blue), of permeable cells and external nucleic acids with propidium iodide (red), and of viable cells using SYTO9 (green).

**FIGURE 7:**
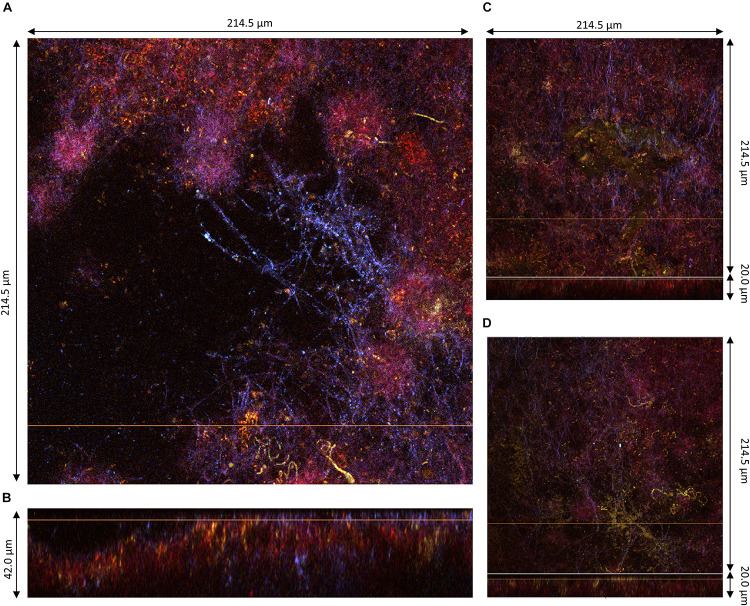
Lower region of pellicles observed at D3 during kombucha elaboration using two-photon microscopy. **(A)** Cross-sections of the bottom surface of sample 5, with the orange line defining the position of the longitudinal section (dimensions: 214.5 × 214.5 μm). **(B)** Longitudinal section of sample 4 (214.5 × 42.0 μm), with the bottom facing up and the orange line defining the position of the corresponding cross-section. **(C,D)** Assembled images of the cross-section on the top and longitudinal sections with the bottom facing up at the bottom of sample 5 (214.5 × 214.5 × 20.0) and sample 6 (214.5 × 214.5 × 20.0), respectively. Fluorescence labeling of cellulose with calcofluor (blue), of permeable cells and external nucleic acids with propidium iodide (red), and of viable cells using SYTO9 (green).

The samples 4, 5, and 6 obtained at D3 were much thicker than those at D2, hence z-stacks ranging between 40 and 143 μm (Figure 6). Samples at D3 clearly conserved their layered organization with a common pattern across samples: a top layer consisting of cellulose fibrils and a sublayer stained by PI containing viable bacteria cells and supposedly yeast debris similar to sample 1 (Figure 3). The top layer had variable thickness from less than 20 μm (Figure 6A) to more than 100 μm (Figures 6B,C). In all samples, viable cells could be observed at the top surface like the samples at D2 (Figures 4, 5), and unstained strata also appear inside the top layer (Figures 6B,C). Despite the good diffusion of fluorescent probes, the PI-stained sublayer appears to act as a barrier for laser penetration, which could be due to its physical property. Therefore, penetration and detection of photons was strongly lowered beyond this layer. So, the pellicles were without a doubt thicker than the z-stacks, hence the double acquisitions made from the top surface (Figure 6) and from the bottom surface (pellicle upside-down on the glass slide) (Figure 7).

The bottom surface of all samples was dominated by PI staining of around 10 μm in thickness and which seemed established in the preexisting bottom cellulose layer (Figures 4B, 5B,D). This liquid/biofilm interfacial region hosted numerous viable bacteria sometimes organized in chains. It seems like PI-stained biomass was trapped by cellulose fibrils. Those fibrils have a different aspect than on the top surface with a spider net pattern rather than parallel, with a compact fur-like aspect (Figures 4A, 6A).

### Comparison of Kombucha Pellicles With Pellicles From Pure Acetic Acid Bacteria Cultures

**FIGURE 8:**
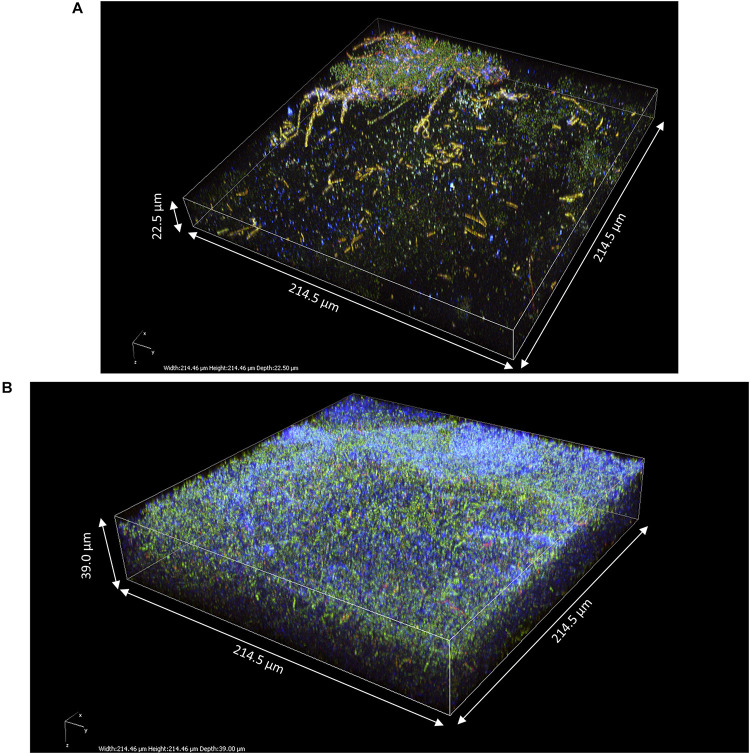
3D modeling of z-stack of the upper region of pure acetic acid bacteria pellicles observed after 14 days of culture in modified sugared tea using two-photon microscopy: **(A)** *Acetobacter indonesiensis*-grown pellicle (214.5 × 214.5 × 22.5 μm) and **(B)** *Komagataeibacter saccharivorans*-grown pellicle (214.5 × 214.5 × 39.0 μm). Fluorescence labeling of cellulose with calcofluor (blue), of permeable cells and external nucleic acids with propidium iodide (red), and of viable cells using SYTO9 (green).

To investigate the impact of yeasts on the structure of pellicles, z-stacks of pure AAB-grown pellicles were also acquired (Figure 8). These pellicles were composed of viable bacteria cells and cellulose fibrils. Overall, the aspect of pure AAB pellicles was much homogenous. The shape of cellulose fibrils of *A. indonesiensis* appeared shorter than that of *K. saccharivorans*. The *A. indonesiensis* cells also tended to arrange more often in chains compared to *K. saccharivorans*. Despite 14 days of incubation, the thickness of those biofilms (22.5 and 39.0 μm; Figures 8A,B, respectively) appeared to be much smaller than those in kombucha at D3 (more than 80 μm) (Figure 6). However, the thickness was more similar to that of kombucha pellicles at D2, which was between 27 and 86 μm (Figures 4, 5). But even with similar thickness, pure culture pellicles showed a poor consistency at a macroscopic scale (Figures 1E,F). When comparing with D2 kombucha pellicles, the striking difference lies in the lack of alternation between the cellulosic layer and the IP-stained middle layer with viable cells and biomass (Figures 4–6). As a result, the absence of yeasts limited the growth rate of the pellicle and led to the absence of the biomass-rich layer. AAB cells can instead be found in any part of the pure culture pellicle. However, the characteristic laminar structure might be observed after a further incubation time since it has been reported in static AAB pure cultures (Gullo et al., 2018). Finally, the results pointed clearly at the role of yeasts as enhancers of pellicle formation and in the early establishment of the biomass-rich layer stained by PI.

## Discussion

The layered structure of kombucha pellicles observed in this study echoes the theoretical structure of the AAB pellicle given by Gullo et al. (2018). Based on the gathering of previous experimental and modeling data, the authors described a putative cellulosic pellicle structure that, to our knowledge, has not yet been confirmed by microscopic observation. This structure is organized in layers, with an active cellulose-producing layer located between 50 and 100 μm under the top surface of the pellicle. The reason of this location can be explained by the evaporative pressure at the very top of the pellicle and an optimal balance between oxygen and substrate gradients, with the latter being the limiting factor after the establishment of the pellicle (Verschuren et al., 2000). The biomass-rich layers hosting viable bacteria cells could be observed in the present study beneath a top-surface cellulosic layer between 10 and 120 μm and seem to fulfill this function. Filamentous cellulose on the bottom surface was also observed at a microscopic scale as mentioned by the above-cited review (Gullo et al., 2018). Generally, the observations made on the kombucha pellicle are coherent with the model proposed for pellicles produced by AAB, but the presence of yeasts clearly impacted the formation of the biofilm.

Based on comparison with pure AAB biofilm grown in sugared tea, yeasts boosted the formation of the pellicle and induced the presence of the heavily PI-stained middle layer that may be caused by the release of nucleic acids and other intracellular material from dead yeast cells, although bacteria could also release plasmids as part of horizontal gene transfers (Van Meervenne et al., 2014). The review of Gullo et al. (2018) also mentions the possibility for this layer to be the support of microbial communication and nutrition. Moreover, yeast aggregates during the very early steps of pellicle formation could play a role of scaffold by supporting the network of cellulose fibrils. It can be suspected that yeast aggregates include filamentous or pseudo-hyphal forms as reported in strawberry vinegar biofilm (Valera et al., 2015), namely, the formation of pseudo-mycelium was reported in wine for *B. bruxellensis* and *S. cerevisiae* and is suspected to be triggered not only by nutritional depletion in carbohydrate and nitrogen in the growth medium but also by metabolites produced by other yeasts such as fusel alcohols (Uscanga et al., 2000; Dickinson, 2008; Louw et al., 2016). The formation of pseudo-mycelium by yeast in the context of kombucha could therefore be linked to the low nitrogen content of sugared tea and/or by microbial interactions that have yet to be determined.

The entrapment of yeasts by bacterial cellulose could benefit AAB by keeping their main source of substrate available in a confined space. Whether entrapped yeasts are alive and provide monosaccharide and ethanol or release assimilable nitrogen through autolysis, they would be used as nutrient storage. This way, the positive action of yeast activity on AAB observed previously in the liquid phase could occur in an optimized space inside the biofilm (Tran et al., 2020a). This would imply that the pellicle, particularly within the active biomass-rich layer, acts as a catalyzer for the organic acid production of AAB occurring during the aerobic acidification phase of kombucha production. This metabolic boost could then also stimulate the production of cellulose during and after the early steps of biofilm formation. Due to the lack of labeled viable yeasts inside the pellicle, it is difficult to conclude on the benefits for yeasts of being embedded in the cellulose matrix in terms of protection against biotic and abiotic perturbation. Nevertheless, research works on the molecular interaction of cellulose show that it is able to bind polyphenols and heavy metals, which could inhibit their toxicity toward microorganisms dwelling in the pellicle (Phan et al., 2015; Najafpour et al., 2019).

## Conclusion

The kombucha biofilm presents similarities to cellulosic pellicles grown by pure AAB in sugared black tea but differs by the presence of yeast, which are involved from the early steps of biofilm formation. Based on observations, the formation model consists in an initial trapping and aggregation of yeasts (possibly in pseudo-mycelium form) in bacterial cellulose. With accumulation of cellulose and cells, a consistent layered pellicle is formed and, from then on, grows in thickness. The top layer at the interface with air is made of parallel cellulose fibrils and hosts yeasts and bacteria, while the bottom surface at the interface with liquid is made of cellulose network colonized by bacteria and where biomass accumulates. Finally, a middle layer located under the top cellulosic layer is filled with biomass and viable bacterial cells that are suspected to be the active agents of pellicle growth. This region is thought to play a nutritional function for bacteria by taking advantage of entrapped yeast metabolism and autolysis, thus revealing an aspect of the microbial interactions in kombucha. This study indicates that the yeast–AAB interactions in kombucha act on the structure and building of the pellicle, which could, in turn, enhance other types of interactions, including the metabolic interplay necessary for optimal kombucha production. The function of the pellicle as a catalyzer of the biological acidification of kombucha could be further investigated to help optimize its production, namely, the method used in this study could benefit from improvements regarding the penetration and concentration adjustments of fluorescent labels. The optimization of laser compensation would also be used to obtain a better signal. Further investigation should focus on the discrimination of acetic acid bacteria from lactic acid bacteria using fluorescence *in situ* hybridization labeling for the determination of localization differences in the structure, for example, regarding the access to oxygen. The contribution of yeasts in exopolymers inside the cellulosic matrix itself could also be of interest using relevant probes.

## Data Availability Statement

The raw data supporting the conclusions of this article will be made available by the authors, without undue reservation.

## Author Contributions

TT took the lead of the writing of the article. PW gave access and support to two-photon microscopy through the Dimacell Imaging Facility. FV and AM provided the kombucha cultures used in the experiments. All authors provided critical and complementary elements to the manuscript.

## Conflict of Interest

The authors declare that the research was conducted in the absence of any commercial or financial relationships that could be construed as a potential conflict of interest.

## Publisher’s Note

All claims expressed in this article are solely those of the authors and do not necessarily represent those of their affiliated organizations, or those of the publisher, the editors and the reviewers. Any product that may be evaluated in this article, or claim that may be made by its manufacturer, is not guaranteed or endorsed by the publisher.

## Footnotes

1
https://blast.ncbi.nlm.nih.gov/Blast.cgi
